# A chronic stress-induced microbiome perturbation, highly enriched in *Ruminococcaceae_UCG-014*, promotes colorectal cancer growth and metastasis

**DOI:** 10.7150/ijms.90612

**Published:** 2024-03-25

**Authors:** Ling Zhao, Xinxin Hou, Yuanyuan Feng, Yingru Zhang, Shiyun Shao, Xinnan Wu, Junfeng (Jim) Zhang, Zhaozhou Zhang

**Affiliations:** 1 School of Integrative Medicine, Shanghai University of Traditional Chinese Medicine, 1200 Cailun Rd, Shanghai 201203, China.; 2 Department of Medical Oncology, Shuguang Hospital Affiliated to Shanghai University of Traditional Chinese Medicine, Shanghai 201203, China.; 3 Department of Traditional Chinese Medicine, Putuo Hospital Affiliated to Shanghai University of Traditional Chinese Medicine, Shanghai 200062, China.; 4 Nicholas School of Environment & Duke Global Health Institute, Duke University, 2080 Duke University Road, Durham, NC 27708, USA.

**Keywords:** Chronic stress, chronic restraint stress, colorectal cancer, metastasis, gut microbiome, gut microbial metabolites, *Ruminococcaceae_UCG-014*.

## Abstract

**Purpose:** Mounting evidence indicates that psychological stress adversely affects cancer progression including tumor growth and metastasis. The aim of this study was to investigate the role of chronic stress-induced microbiome perturbation in colorectal cancer (CRC) progression.

**Methods:** Chronic restraint stress (CRS) was used to establish the chronic stress mouse model, behavioral tests were used for the CRS model evaluation. Subcutaneous xenograft model and lung metastasis model were established to investigate the growth and metastasis of CRC promoted by CRS exposure. 16S rRNA gene sequencing and liquid chromatograph-mass spectrometer (LC-MS) were applied to observe the effects of CRS exposure on the alteration of the gut microbiome and microbial metabolites. Bioinformatics analysis and correlation analyses were applied to analyse the changes in the frequency of body mass, tumor volume, inflammatory factors, neuroendocrine hormones and metabolites of the gut microbiota.

**Results**: In this study, we identifed that CRS exposure model was appropriately constructed by achieving expected increases in disease activity index and enhanced depressive-like behaviors. CRS exposure can promote growth and metastasis of CRC. Besides, the data indicated that CRS exposure not only increased the neuro- and immune-inflammation, but also weakened the gut mucosal immunological function. The 16s rRNA gene sequencing data showed that CRS exposure increased the abundance of *g_Ruminococcaceae_UCG_014*. Furthermore, the LC-MS data indicated that with only 2 exceptions of carpaine and DG (15:0/20:4(5Z,8Z,11Z,14Z)/0:0), the majority of these 24 metabolites were less abundant in CRS-exposed mice. Bioinformatics analysis and correlation analyses indicated that only *Ruminoscoccaceae-UCG-014* was significantly associated with inflammation (IL-6), neurotransmission (5-HT), and microbial metabolism (PS).

**Conclusion:** CRS exposure altered diversity, composition and metabolites of the gut microbiome, with *Ruminococcaceae_UCG-014* perturbation consistently correlated to inflammatory responses, suggesting a particular role of this bacterial genus in CRC growth and metastasis.

## Introduction

A cancer diagnosis is a life-changing event, commonly accompanied with psychological stress and depression 1. Psychological stress is a multi-factorial unpleasant experience of a psychological, social, spiritual, and/or physical nature that may interfere with one's ability to cope effectively with cancer, physical symptoms and treatment 2. Chronic stress can induce depressive behaviors in mice, characterized by reduced food intake and activity, as well as abnormal behavioral tests 3. Epidemiological studies have established a significant association between psychological stress and mortality of cancer including colorectal cancer 4. Some studies reveal that psychological stress adversely affects cancer progression including tumor growth, metastasis, and cancer-treatment efficacy 5, 6. The pathophysiologic mechanisms underlying this association, however, remain poorly understood, despite recent findings that CRC correlates with alterations in microbiota composition 7 and that CRC alters the gut microbiota activity 8 and regulates expression of the gut microbiome to adapt to changes in environmental conditions 9.

Colorectal cancer is the third most prevalent cancer worldwide 10. The colon and rectum are the main sites where most of the gut microbiota colonizes, inhabits, and interacts with the host. Known as “the second fingerprint” or “the second genome” of humans 11, the gut microbiota participates in various physiological functions such as digestion, metabolism, immune regulation and intestinal mucosal defense. Under normal physiological conditions, the gut microbiota is in a relative dynamic equilibrium with the host and the external environment. Mounting evidence indicates that psychological stress disrupts the balance of gut microbiota by reducing microbial diversity and altering the structure of gut microbiota 12, 13. However, a mechanistic link has yet to be established between stress-induced perturbation of the gut microbiome and the progression of colorectal cancer. Such a link is important to understand whether and how psychological stress promotes tumor growth and metastasis. Moreover, identifying stress-affected bacteria and microbial metabolic pathways, in relation to cancer progression, will aid future treatment strategies. This study was designed to investigate the role of chronic stress-induced microbiome perturbation in CRC progression, which may help find a novel therapeutic target.

## Materials and methods

### Chronic restraint stress mouse model

**Figure 1:**
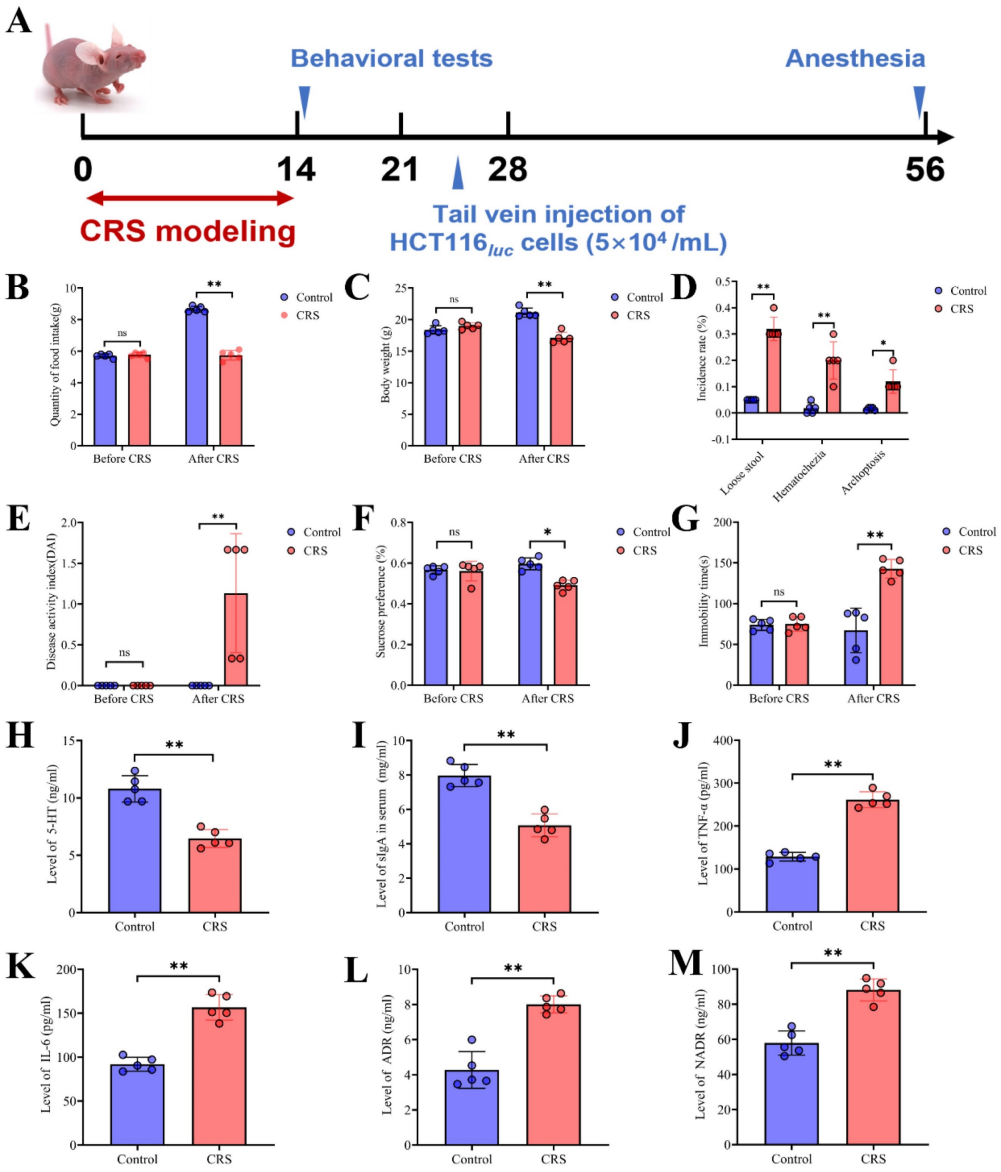
Construction and evaluation of the CRS model. **A** Schematic showing the time period of CRS exposure, the time period for behavioral tests, and the time point for the inoculation of human colon cancer cells (HCT116). From Days 0 to 14, CRS mice were placed in a 50ml tube for 8 hours per day. Control mice did not receive CRS but underwent the same timeline and procedures for HCT inoculation and tests. **B** average food intake before and during the CRS period (n=5 for each of the CRS and control group); **C** average body weight before and during the CRS period (n=5 per group); **D** incidence for loose stool, hematochezia and archoptosis, measured at the end of 14-day CRS exposure (n=5 per group); **E** disease activity index (DAI) scores measured before and at the end of CRS exposure (n=5 per group; the DAI score includes scales for stool consistency, body weight loss, fur texture, and animal posture). **F** sucrose preference (%) measured before and at the end of CRS exposure (n=5 per group); **G** immobility time measured before and at the end of CRS exposure (n=5 per group). **H** serum concentrations of 5-HT measured (n=5 per group); **I** expression of sIgA in colorectal tissues (n=5 per group); **J**, **K**, **L** and **M** serum concentrations of TNF-α, IL-6, ADR, and NADR, respectively (n=5 per group). Data are represented as mean±SEM from triplicates of three independent experiments, asterisks indicate significant difference between CRS and control mouse groups: ^*^P<0.05, ^**^P<0.01, ^***^P<0.001.

Six-aged male BALB/c nude mice (20±2g) were purchased from Sino-British SIPPR/BK (Shanghai, China). Mice were maintained in a specific pathogen-free environment under a 12-hour light-dark cycle, a temperature of 22 °C, and relative humidity of 60 % during the experiment, with access to food and water ad libitum. After 1-week acclimatization, mice were randomly assigned to the control group and the CRS-exposure group (n=10/group). CRS was performed according to previously published protocols 14, 15. Mice in the CRS group were restricted in a 50ml tube with ventilation holes for 8h per day (from 9:00 to 17:00) and 14 consecutive days commencing 10 days before tumor cell inoculation. Control mice were maintained in their home cages. The experimental schedule is shown in Fig. 1A**.** Throughout the experiments, general health conditions, body mass, food intake, stool status and the disease activity index (DAI) were regularly monitored every two days throughout animal experiments. One week after the start of CRS exposure, two behavioral tests were performed as described below.

### Sucrose preference test

The SPT (sucrose preference test, SPT) was performed according to previously published protocols 14. The 2-bottle choice procedure was used to investigate anhedonia. First of all, mice were allowed to acclimate for 7d under the 2-bottle choice procedure conditions. 1% sucrose (SIGMA-ALDRICH) diluted in reverse osmosis (RO) water or RO water alone was filled into the 50-mL tubes with stoppers fitted with ballpoint sipper tubes. With the 2 inverted tubes placed into a wire lid, both bottles were placed on one side of the divided wire rack, leaving the other side for food. The weights of solutions were recorded. The tubes were rotated daily to avoid false sucrose preferences caused by side bias. An estimate of sucrose preference was derived by dividing sucrose intake by total fluid consumption. The volume formula was applied to determine consumption, and the volume of a cylinder equation was: V=πr^2^h, where V =volume (in cm^3^), π=3.1416, r=interior radius (in cm), and h=distance between daily markings (in cm).

### Tail suspension test

The tail suspension test (TST) was conducted as previously described 16. Briefly, mice were suspended 60 cm above the floor with adhesive tape placed approximately 1 cm from their tail tips. A investigator blind to the study recorded the duration of immobility within six minutes. Mice were considered immobile only when hung passively and were motionless. Any mice climbing their tails were excluded from the experiment.

### Subcutaneous xenograft model of colorectal cancer

After the CRS exposure and behavioral tests, 10 mice in total were included from the experiments and thus, there were 5 mice for each of the CRS and control group. Human colon cancer HCT116 cells were obtained from the cell bank of Shuguang Hospital Affiliated to Shanghai University of Traditional Chinese Medicine. HCT116 cells were cultured and passaged conventionally with Dulbecco's Modified Eagle Medium (DMEM, Gbico, USA), supplemented with 10% fetal bovine serum (FBS, Sigma, USA), 100 units/ml of penicillin-streptomycin (Invitrogen, Carlsbad, CA) at 37°C. When the cell growth density in the culture flask reached 80%, cells were harvested and diluted in PBS, and then injected subcutaneously (2×10^6^ cells per 100 μL per mouse) into the mice's dorsal skin (Shanghai Sino-British SIPPR/BK Laboratory Animal Co., Ltd), the protocol was referred to published studies 17, 18. On the 7th day after subcutaneous xenograft, the tumor volume was measured using a vernier caliper every 3 days. The tumor volume was calculated using the following formula: volume (mm^3^)=1/2×length×(width)^2^. Finally, experimental mice were sacrificed, and the tumors were dissected. The humane endpoints of the study include rapid weight loss (lose 10%-20% of the body weight within a week), loss of appetite (The appetite level less than 50% of the normal amount, loss of appetite for 24 hours, or poor appetite for 3 or more days), No eating and drinking behaviors, oversize of tumor growth (Tumor exceeds 10% of the animal's body weight, and the average tumor diameter exceeds 15mm) and tumor rupture (tumor grows rapidly to fester, causing infection or necrosis). During the modeling process, no mouse meets the criteria of humane endpoints. All mice were euthanized finally at the end of the experiment. In order to minimize the fear, anxiety, and pain of experimental animals, we adopted the euthanasia method of excessive CO_2_ inhalation.

### Lung metastasis model of colorectal cancer

Human colon cancer HCT116 cells transduced with lentiviral vectors transfer of luciferase were prepared. Cells were grown to 80% confluency in 60mm dish. HCT116*_luc_* cells were cultured in Dulbecco's Modified Eagle Medium (DMEM, Gbico, USA) containing 10% fetal bovine serum (FBS) and 100 units of penicillin-streptomycin (Invitrogen, Carlsbad, CA) in a humidified incubator at 37°C with 95% air and 5% CO_2_. When the HCT116*_luc_* cell growth density in the culture flask reached 80%, cells were harvested and diluted in PBS at a final concentration of 5×10^7^cells/ml. Then a 0.2 ml of the cell suspension containing 5×10^4^ cells/ml was injected into the tail vein. Bioluminescence imaging was applied to monitor the tumor development of lung metastasis.

### Bioluminescence imaging

Bioluminescence imaging was conducted using bioluminescence technology (Night OWLII LB983 NC100, Berthold, Germany). Before imaging, all mice received 2.5mg of D-luciferin (Xenogen Corporation, Hopkinton, MA) intraperitoneally. Mice receiving the tail vein injections were imaged weekly in this same way to monitor the condition of tumor growth.

### Hematoxylin-eosin staining (HE)

Mice were initially anesthetized with diethyl ether and sacrificed by dislocation, both the subcutaneous xenograft tumor tissues and the lung metastatic tumor tissues were prepared by routine HE. After fixing with 10% formaldehyde for 24 hours, tissue was dehydrated, permeabilized, wax-dipped, paraffin-embedded, and cut into 3 μm sections. Slices were stained with HE Staining Kit (Solaribio, Beijing) referring to the manufacturer' instruction.

### Immunohistochemistry (IHC)

Microvascular density (MVD) was detected by IHC and determined by counting microvessels in selected fields. The tissue slides were deparaffinized with xylene and rehydrated with a gradient concentration of ethanol. Antigens were retrieved by boiling under pressure in EDTA buffer (pH=9.0) for 3 minutes. After incubating with 0.3% H_2_O_2_ for 20 minutes, the sections were blocked for 45 minutes with goat serum before being washed with PBS, and then incubated with primary antibodies at 4 °C overnight. The next day, sections were incubated with the secondary antibody at 37 °C for 30 minutes, followed by the HRP-labeled streptavidin solution for 30 minutes. Sections were visualized following a 5 min incubation of 3, 3-diaminobenzidine-tetrachloride (DAB) and a counterstain of hematoxylin. Semi-quantitative analysis was performed using Image Pro Plus 6.0.

### Enzyme-linked immunosorbent assay (ELISA)

Serum concentrations of IL-6, TNF-α, 5-HT, ADR and NADR were analyzed using mouse ELISA kits (Bogoo, Shanghai, China) according to the manufacturer's instructions. The optical density was resulted from a microplate reader (SpectraMax 190, Molecular Devices, USA) at 450 nm wavelength. Colonic secretory immunoglobulin A (sIgA) was also analyzed with a mouse ELISA assay kit (Bogoo, Shanghai, China).

### DNA extraction and V3 and V4 regions of 16S rRNA gene sequencing

The faecal samples were collected in the morning (8:00 to 9:00 a.m.) under sterile conditions before all the experimental mice were sacrificed. Total bacterial DNA was extracted from mouse fecal sample with the E.Z.N.A.® soil DNA Kit (Omega Bio-tek, Norcross, GA, U.S.) according to manufacturer's instructions. The purity and concentration of the DNA extracts were determined with NanoDrop 2000 UV-vis spectrophotometer (Thermo Scientific, Wilmington, USA). The V3-V4 region of 16S rRNA were amplified by an ABI GeneAmp® 9700 PCR thermocycler (ABI, CA, USA) with the primer pairs 338F (5'-ACTCCTACGGGAGGCAGCAG-3') and 806R (5'-GGACTACHVGGGTWTC TAAT-3'). H instead of A/C/T bases, V instead of A/G/C bases and W instead of A/T bases. Purified amplicons were pooled in equimolar and paired-end sequenced (2×300) on an Illumina MiSeq platform (Illumina, San Diego, USA) according to the standard protocols by Majorbio Bio-Pharm Technology Co. Ltd. (Shanghai, China). The raw reads were deposited into the NCBI Sequence Read Archive (SRA) database. The raw sequencing reads were demultiplexed, filtered and then merged. Operational taxonomic units (OTUs) with 97% similarity cutoff (Liu et al. 2017) were clustered using UPARSE (version 7.1, http://drive5.com/uparse/), and chimeric sequences were identified and removed. The taxonomy of each OTU representative sequence was analyzed by RDP Classifier (http://rdp.cme.msu.edu/) against the 16S rRNA database (eg. Silva SSU128) using confidence threshold of 70%.

### Analysis of microbial metabolites

Frozen fecal samples of individual mice were thawed at room temperature. A 50 mg fecal aliquot was prepared with 400 μL methanol-water (4:1, v/v), and homogenized for 10 seconds. Then, fecal suspension was ultrasonically extracted on ice for 10 minutes and held at -20 °C for 30 minutes, centrifuged for 15 minutes at 13000 rpm at 4 °C. The supernatant was injected into liquid chromatograph-mass spectrometer (LC-MS). Quality control (QC) samples were prepared by mixing aliquots of all analytic samples into a pooled sample, which was analyzed using the same method as the analytic samples for assessing repeatability of the analytical method. The supernatants were analyzed with an Acquity BEH C18 column (100mm×2.1 mm i.d., 1.7µm; Waters, Milford, USA) based on AB Sciex TripleTOF 5600TM mass spectrometer system (AB SCIEX, USA). The metabolites were separated with two mobile phases, phase A was the mixture of acetonitrile and isopropanol (1:1 v/v) contained 0.1% formic acid and phase B was water contained 0.1% formic acid. The elution gradient was set as follows: 5% B-30% B over 0-3 min, 30% B-95% B over 3-9 min, 95% B-95% B over 9-13.0 min; 95% B-5% B over 13.0-13.1 min, and 13.1-16 min holding at 5 % B at a flow rate of 0.40 mL/min. Injection Volume was 2 μL. The mass spectrometric data were collected using an AB Sciex TripleTOF 5600TM mass spectrometer system equipped with an electrospray ionization (ESI) source in either positive or negative ion mode with a capillary voltage 1.0 kV, sample cone, 40 V, collision energy 6 eV. The source temperature was set at 120^°^C, with a desolvation gas flow of 45 L/h. Centroid data were collected from 50 to 1000 m/z with a 30000 resolution.

### Bioinformatics analysis

Principal component analysis (PCA) was used to identify trends and patterns of our high-dimensional data. Principle coordinate analysis (PCoA) was applied to profile different distance metrics (e.g., Bray-Curtis distances). These metrics were used, complementary to the Euclidean distance metric generated in the PCA. Both PCA and PCoA were conducted using the Majorbio I-Sanger Cloud platform (www.i-sanger.com).

### Correlation analyses

The relative abundances of bacteria were correlated with changes in the frequency of body mass, tumor volume, inflammatory factors, neuroendocrine hormones and metabolites of the gut microbiota. Correlation coefficients and the p-values (set to 0.05 for significance) were calculated using the Pearson parametric correlation test using the R package “ggcorrplot”. Correlational circus maps were generated using the R package “igraph”. Pathway analysis and biological function enrichment analysis were based on the Kyoto Encyclopedia of Gene Genotype (KEGG) enrichment using the R package “DOSE”, “GO.db”, “GSEABase” and “ClusterProfiler”. Only pathways with a false discovery rate (FDR) corrected p-value of<0.05 were represented. The correlation coefficients had a value range of (-1, +1). The coefficients are presented in the form of a heat map with color gradients reflecting the degree of correlations.

### Statistical analysis

All measurement data were presented as mean±standard deviation (SD) of three independent measurements. The unpaired two-tailed Student's t-test was applied in two-group comparisons. Based on the online platform of the Majorbio I-Sanger Cloud, the difference of gut bacteria was analyzed with the Wilcoxon rank-sum test. The *P*-value was based on a two-tailed test with FDR corrected and the 95% confidence intervals were calculated through the bootstrap algorithm. Statistical significance was set at *P*<0.05.

## Results

### Evaluation of the CRS model and neuro-inflammatory levels

CRS exposure was imposed to mice by restricting them in a 50ml tube with ventilation holes for 8 hours per day throughout 14 consecutive days (Fig. 1A). We evaluated this model by comparing the CRS group with control (i.e., mice without CRS exposure) for disease activity index and depressive-like behaviors. We found that CRS exposure resulted in significant decreases in food intake and body weight (Fig. 1B&1C). Following the 14-day CRS exposure, mice exhibited stool abnormalities, as reflected in significantly increased incidence of loose stool, hematochezia and archoptosis, respectively (Fig. 1D). Moreover, following CRS exposure, depressive-like symptoms were significantly worse compared to control mice, as reflected in increased disease activity index derived from stool consistency, body weight loss, fur texture, and animal posture (Fig. 1E), decreased preference of sucrose solution (Fig. 1F), and increased immobility time (Fig. 1G). These observations support that the CRS exposure model was appropriately constructed by achieving expected increases in disease activity index and enhanced depressive-like behaviors. Moreover, we observed a significantly reduced level of sIgA in the colorectal tissues of CRS-exposed mice (Fig. 1I), indicating that CRS exposure weakened the gut mucosal immunological function. Neuro- and immune-inflammation have been shown to affect the integrity of the intestinal structure 19. Hence we measured serum concentrations of neuroendocrine hormones and pro-inflammatory cytokines. We found that compared to control mice, CRS-exposed mice had significantly lower levels of 5-hydroxytryptamine (5-HT or serotonin, a monoamine neurotransmitter) (Fig. 1H) and significantly higher levels of TNF-α and IL-6 as well as stress-related hormones including adrenaline (ADR) and norepinephrine (NADR) (Fig. 1J-1M)**.** These results indicated that CRS exposure inhibited the production of 5-HT and induced the release of stress-related hormones and inflammatory cytokines into the circulatory system, reflective of heightened neuro-inflammatory responses.

### Growth and metastasis of colorectal cancer promoted by CRS exposure

**Figure 2:**
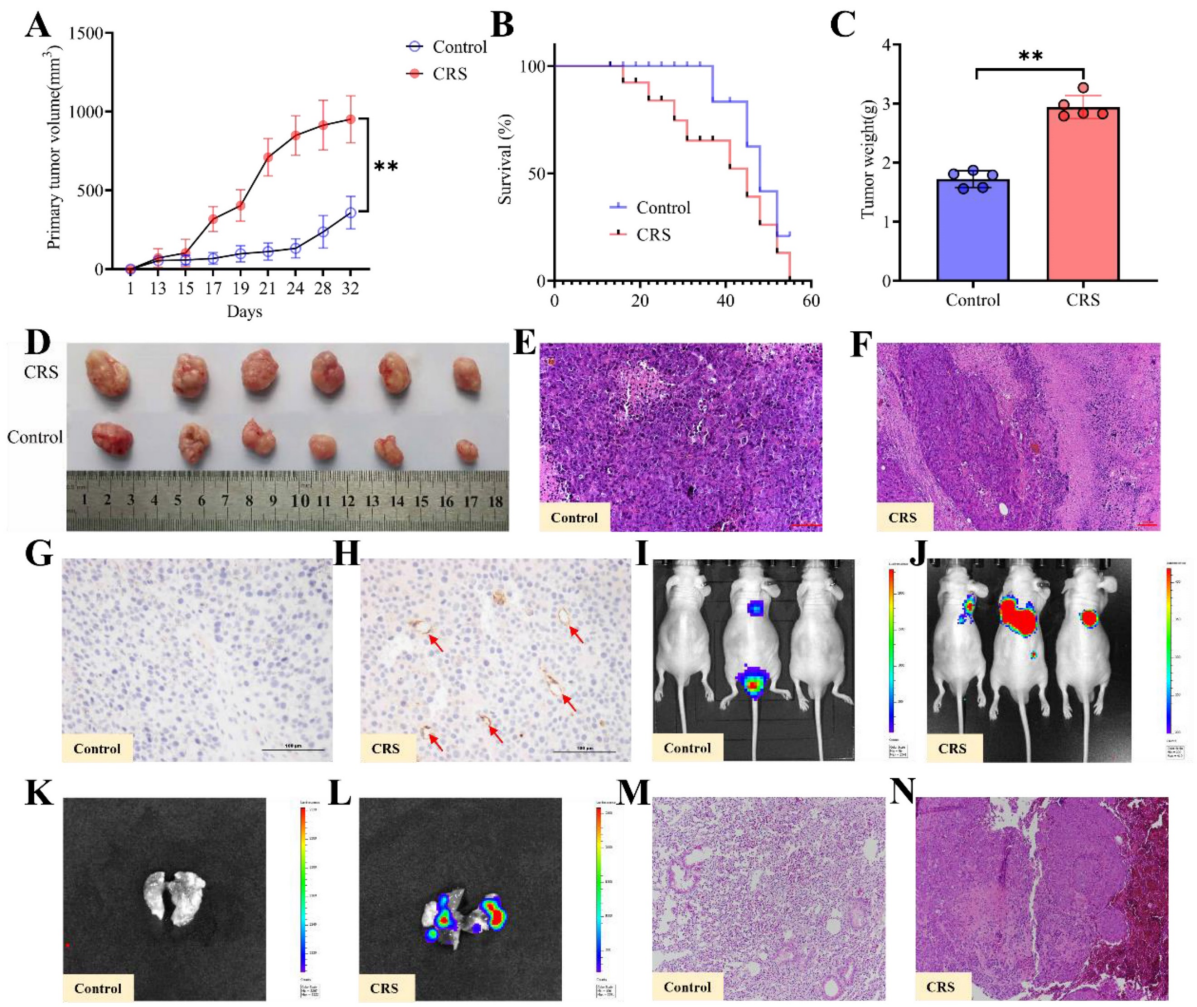
Effects of CRS on the growth and metastasis of colorectal cancer, comparing mice with CRS exposure and mice without CRS exposure (control). **A** primary (subcutaneous colon) tumor volume (length×width^2^×0.5) measured daily (n=5 per group). **B** overall survival rate (n=5 per group); **C** primary tumor weights (n=5 per group); **D** images of primary tumors (n=5 per group); **E** and **F** HE staining of primary tumor tissues; **G** and **H** MVD detection of primary tumor tissues; **I** and **J** lung metastasis of colorectal cancer *in vivo*, where the lung metastasis regions were labelled with red-green fluorescence intensity. The redder the color and the larger the fluorescence area, the greater the lung metastasis; **K** and **L** lung metastasis of colorectal cancer *in vitro*; **M** and** N** HE of lung metastatic tumor tissues. Asterisks showing a significant difference between CRS and control groups: ^*^P<0.05, ^**^P<0.01, ^***^P<0.001.

After the CRS exposure and behavioral tests, human colon cancer HCT116 cells were injected subcutaneously into the nude mice's dorsal skin. With the same timeline and the same protocol, control mice received the same dose of cancer cells (see Methods for details). Both tumor volumes, tumor weights measures and images of primary tumors showed that CRS-exposed mice had significantly larger tumors than control mice (Fig. 2A, 2C & 2D). Starting at Day 18 through Day 55, the overall survival rate was consistently lower for the CRS group than the control group (Fig. 2B). HE staining of the primary tumor tissues indicated that the infiltration of inflammatory cells was more visible in CRS-exposed mice (Fig. 2E&2F**)**. Tumor tissue microvascular density (MVD) detection showed that blood vessels were clearly more visible within the tumor tissues from CRS-exposed mice (Fig. 2H) than those from the control mice (Fig. 2G), indicating that CRS exposure promoted tumor growth by enhancing neovascularization. Using the small animal *in vivo* (Fig. 2I&2J) and *in vitro* (Fig. 2K&2L) imaging technique, we observed not only a significantly higher incidence of lung metastasis in CRS-exposed mice, but also a greater area of the lung tissue affected by the metastasis. HE staining of lung metastatic tumor tissues indicated that the tumor cells from CRS-exposed mice had irregular nucleus, deeper nuclear staining, prominent nucleoli, increased nuclear-to-plasma ratio, and increased capillary network (Fig. 2M&2N).

### Alteration of the gut microbiome by CRS exposure

**Figure 3:**
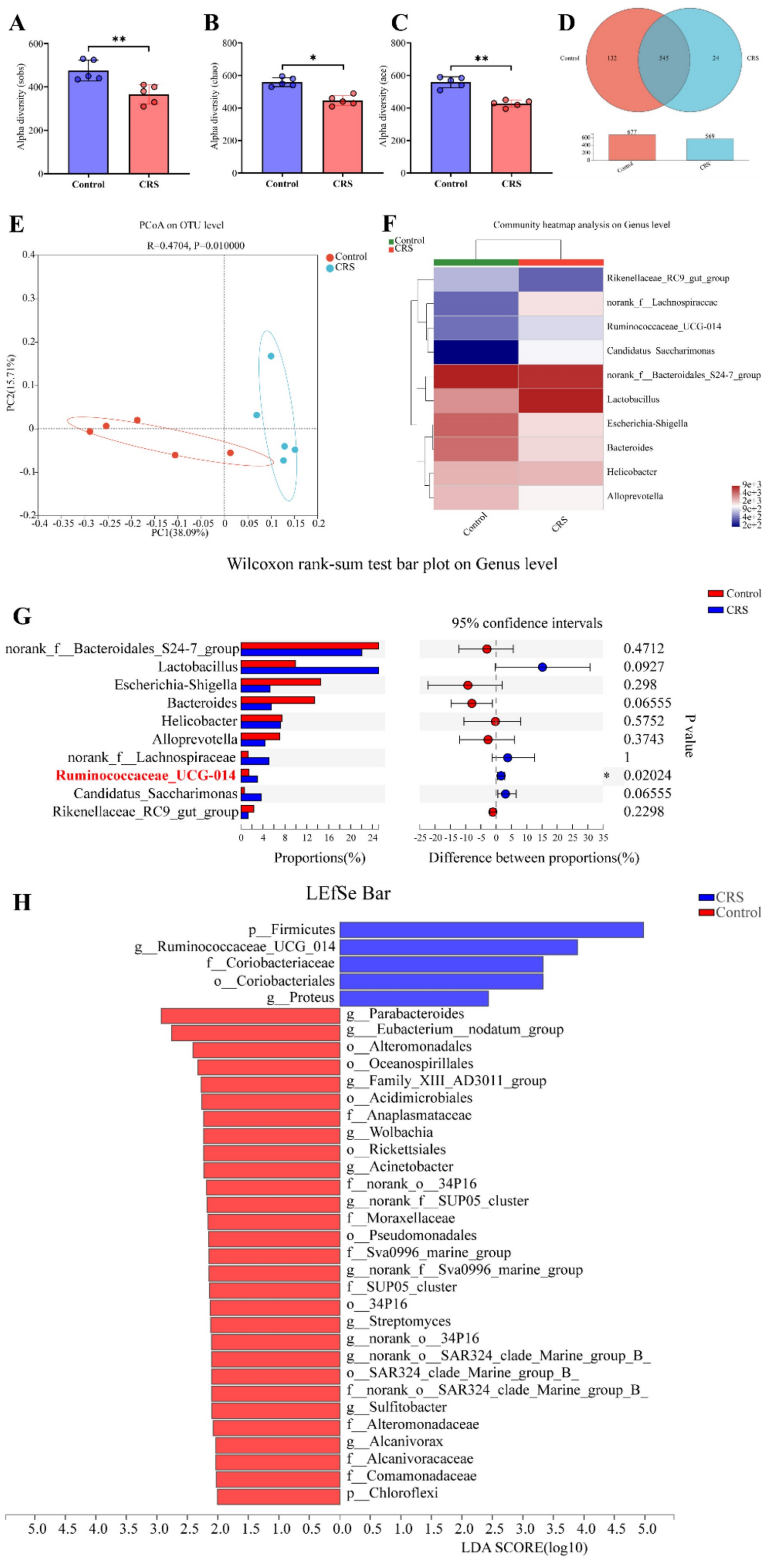
Effects of CRS on gut microbial diversity and microbiota compositions, comparing CRS-exposed and control mice. **A**-**C**, Alpha diversity analyses of the gut microbiota on the operational taxonomic unit (OUT) level using Student's t-test (n=5 per group, * P<0.05); **D** Venn graph showing numbers of bacteria on the OTU level. The number in the overlapping part of the circles represent the number of bacteria shared by both the control (red) and CRS (blue) mice, whereas the numbers in the non-overlapping parts represent the number unique to each mouse group. Bar graph shows total number of OTU bacteria by each group. **E** Plot of principal coordinates analysis (PCoA) on the OTU level based on unweighted-unifrac distance (*R*=0.4704, *P*=0.01000), where the triangles and dots represent stool samples from CRS and Control, respectively, and the X-axis and Y-axis represent the two selected main principal coordinates. **F** heat map from a community compositional analysis, showing the top 10 microbial groups in terms of abundance at the genus level. Relative abundance in the scale of 0 (lowest) to 4 (highest) is shown through color gradient; **G** proportions (%) of a genus in total microbes and differences between CRS and control mice by Wilcoxon rank-sum test (n=6 in each group, *P<0.05); **H** Linear discriminant analysis effect size (LEfSe) showing the microbial groups with community abundance differentiated between CRS and control mice, based on a Linear Discriminant Analysis (LDA) score of≥2.

The 16s rRNA gene sequencing data showed that CRS exposure significantly reduced community diversity of the gut microbiota and altered microbial compositions. On a multi-species level, we identified 34 bacteria that had significant differences in community abundance between the CRS-exposed and control mice. We conducted Alpha diversity analyses on the operational taxonomic unit (OTU) level. Sobs index of community richness, Chao index of community richness, and Ace index of community diversity were all significantly higher in control mice, indicating that CRS exposure reduced community diversity of the gut microbiota (Fig. 3A-C). A Venn OTU analysis of microbiota compositions showed that the two-mouse groups shared 545 common species. However, the number of species unique to the control group (n=132) was substantially greater than the number of species unique to the CRS group (n=24). Overall, the total number of bacteria on the OTU level was 16% smaller in CRS-exposed mice than in control mice (569 versus 677) (Fig. 3D). We conducted a beta diversity analysis to compare microbial compositions on the OTU level across CRS-exposed and control mice using a principal coordinate analysis (PCoA). The two main principal components, representing 53.80% of total microbial factor loadings (38.09% from PC1 and 15.71% from PC2), are plotted with each group's relative contribution to each principal component (PC) (Fig. 3E). This analysis showed that the two groups had distinct distributions across PC1 and PC2, each representing different microbial compositions. Through a community compositional analysis on the genus level, we identified 10 most abundant genera.

Among these, 4 genera (*norank_f_Lachnospiraceae, Resminococcaceae_UCG_014, Candidatus_Saccharimonus, and Lacyobacillus*) appeared to be more abundant in CRS-exposed mice, whereas another 4 genera (*Ricenellaceae_RC9_gut_group, Escherichia_Shigella, Bacteroides, and Alloprevotella*) appeared to be more abundant in control mice (Fig. 3F). However, the abundance of these genera in total number of microbes was significantly different only for *Resminococcaceae_UCG_014* (*P*=0.02024) between the two mouse groups (Fig. 3G). Finally, we conducted a multi-level species Linear Discriminant Analysis (LDA), in which we used a threshold of 2 of linear discriminant analysis effect size (LEfSe). We identified 34 microbial groups that had significant differences in community abundance between CRS-exposed and control mice. Among these 34, only 5 groups showed increased abundance (*p_Firmicutes, g_Ruminococcaceae_UCG_014, f_Coriobacteriaceae, o_Coriobacteriales, and g_Proteus*) and the rest showed decreased abundance in CRS-exposed mice (Fig. 3H).

### Altered metabolisms of the gut microbiota by CRS exposure

**Figure 4:**
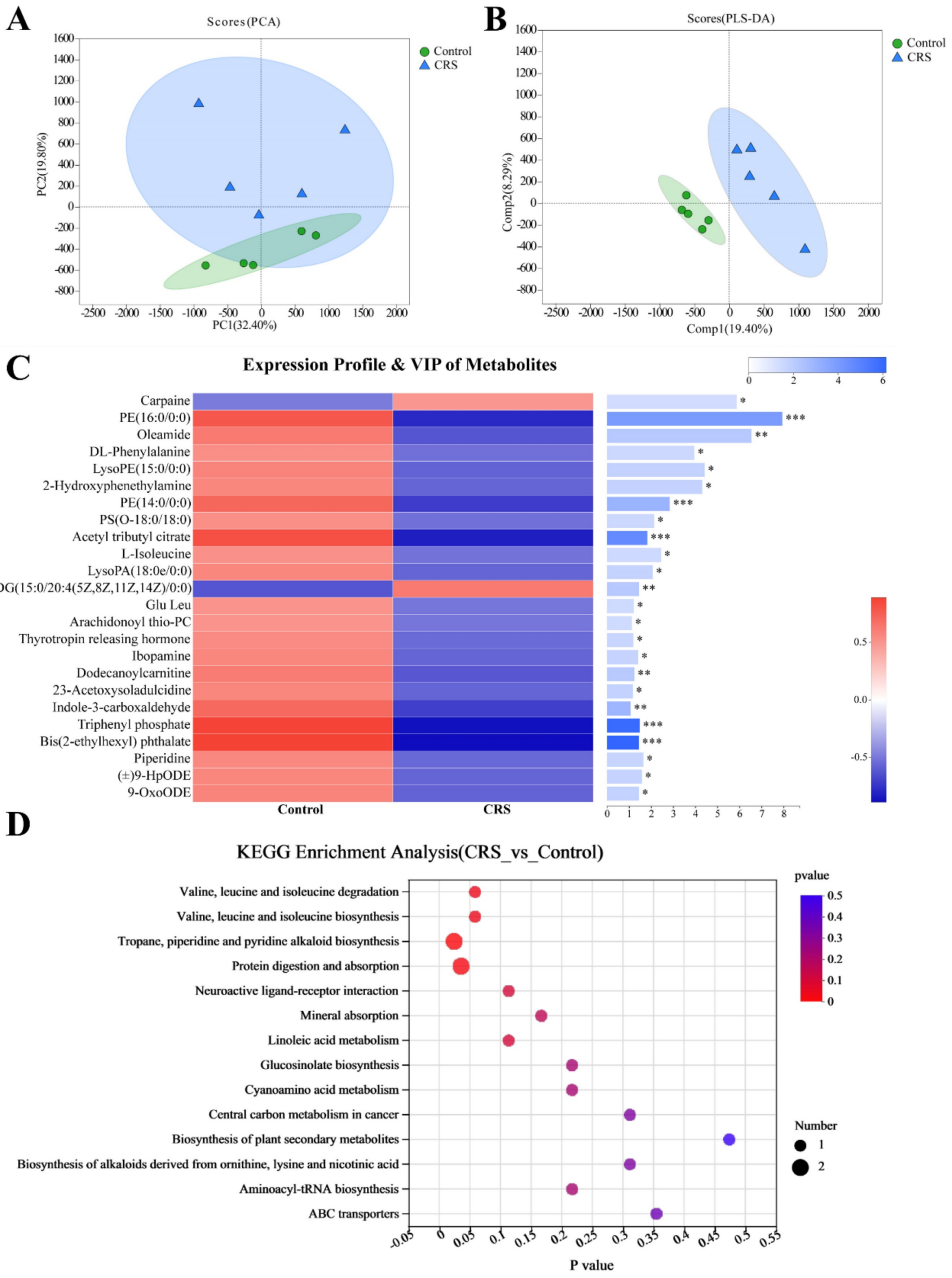
Effects of CRS on gut microbial metabolites, comparing CRS-exposed and control mice. **A** Plot of principal component analysis (PCA) showing the closeness of each sample in CRS or control mice to PC1 and PC2, each representing a unique set of metabolites. The closer the distance between data points, the higher the similarity between samples. **B** Results from Partial Least Squares Discriminant Analysis (PLS-DA) in which Welch's difference test was used and the PLS-DA confidence was 0.95. Comp1 and Comp2 are the two main principal components; **C** Expression profiles and variable importance in the projection (*VIP* values) of the metabolites with differential abundance between the CRS and control groups ( *VIP*≥1 and FDR-adjusted *P*<0.05). The left panel represents relative abundance of the metabolite, whereas the right panel shows VIP values for the metabolite and statistical difference between the mouse groups (^***^ adjusted *P*<0.001, ^**^ *P*<0.01, and ^*^ *P*<0.05); **D** Enrichment ratios specific to metabolic pathways derived from KEGG enrichment analysis, in which enrichment ratio is defined as the number of the metabolites enriched in the pathway divided by the background number of the metabolites annotated to the pathway. P values are FDR adjusted and for differences in pathway enrichment between the CRS and control groups, based on Wilcoxon rank-sum test (* denotes *P*<0.05).

Similar to the PCoA analysis for the community abundance, a principal component analysis (PCA) was applied to metabolites identified by a high-resolution LC-MS technique. Data from individual's samples were plotted showing relative distances among the individual samples and to the two main principal components (PCs), together representing 52.2% of total metabolites (32.40% by PC1 and 19.80% by PC2) (Fig. 4A). Despite some overlap between the data from the two mouse groups, the metabolites were more similar within a mouse group than between the groups. The between-group difference in metabolites was more evident in a Partial Least Squares Discriminant Analysis (PLS-DA), showing within-group distinct clustering of data without between-group overlapping (Fig. 4B). To further examine differences in specific metabolites, we attempted to identify the metabolites meeting the following criteria for between-group differences. Metabolites were selected based on Variable Importance in the Projection (VIP value) >1.0 from the normalized peak intensity. Metabolites were further selected by Student's t-test with a threshold of *P*<0.05 adjusted by a Hochberg-Benjamini-based false discovery rate (FDR). This resulted in 24 differential metabolites between the two mouse groups (Fig. 4C, right panel). With only 2 exceptions of carpaine and DG (15:0/20:4(5Z,8Z,11Z,14Z)/0:0), the majority of these 24 metabolites were less abundant in CRS-exposed mice (Fig. 4C, left panel). Finally, we conducted a KEGG enrichment analysis to estimate enrichment ratios specific to a metabolic pathway. Among the 14 KEGG pathways shown in Fig. 4D, the following 2 pathways were differentially enriched (FDR-adjusted *P*<0.05) between the two mouse groups: the protein digestion and absorption (*P*=0.0354) and the tropane, piperidine and pyridine alkaloid biosynthesis (*P*=0.0241).

### Correlations of bacterial genera with body weight, tumor volume, inflammatory cytokines, neuroendocrine hormones, and microbial metabolites

**Figure 5:**
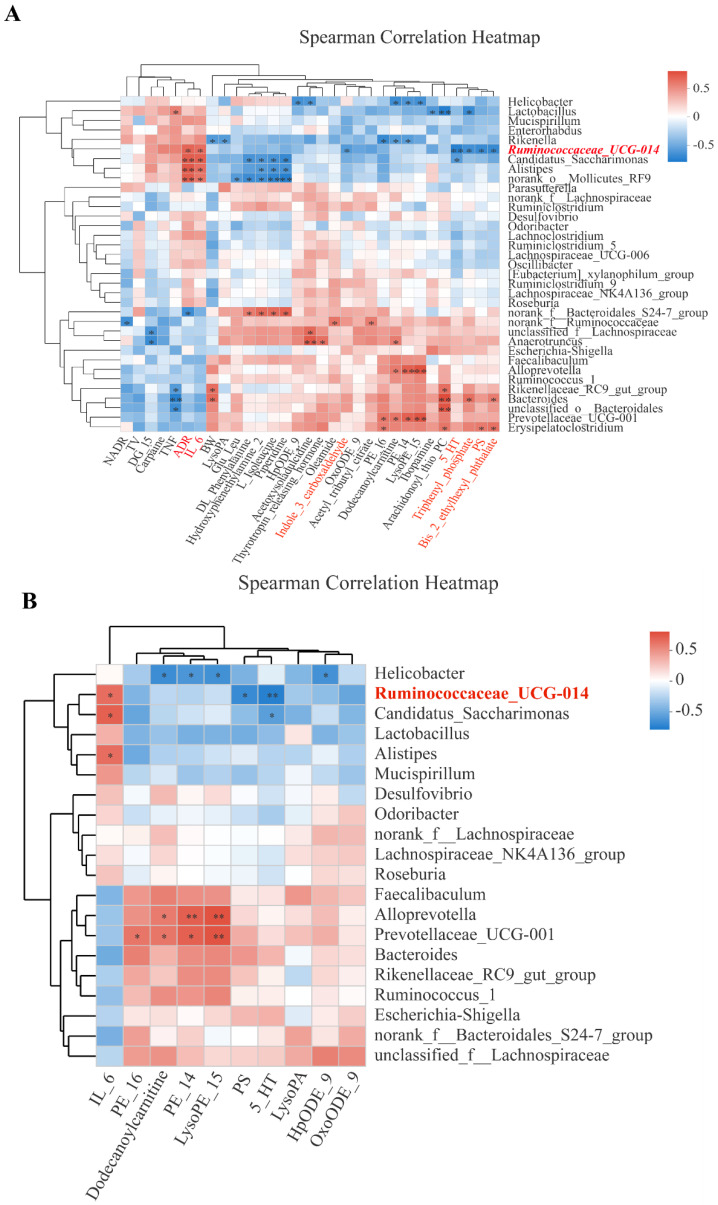
Correlations of bacterial genera with CRS-affected variables. **A** Overview heatmap showing correlations of bacterial genera (Y-axis) with 31 variables including body weight (BW), primary tumor volume (TV), pro-inflammatory cytokines (IL-6, TNF-α), neuroendocrine hormones (ADR, NADR, and 5-HT), and 13 microbial metabolites; **B** Heatmap showing selected correlations meeting Variance Inflation Factor (VIF)<10 and *P*≤0.05 in a PERMANOVA analysis. In both **A** and **B**, Y-axis and X-axis variables are presented by clusters of the variables based on inter-variable correlations. Correlation coefficient (R) between a bacteria genus and an X-axis variable is reflected in color gradients. Statistical significance is denoted as ^*^ for *P*≤0.05 and ^**^ for *P*≤0.01.

Among the bacterial genera shown on the Y-axis, various clusters were formed based on their inter-correlations. Likewise, clusters were formed for the X-axis variables. In the 'overview' heatmap (Fig. 5A), we found that body weight (BW) was significantly correlated with three genera (*Rikenella, Rikeenellacae_RC9_gut_group, and Bacteroides*). Two pro-inflammatory cytokines (IL-6 and TNF-α), clustered with a stress hormone (ADR), were significantly correlated with *Ruminococcaceae_UCG-014* and three closely clustered genera. Neurotransmitter 5-HT was clustered with 3 microbial metabolites (Triphenyl_phosphate, PS, and Bis_2_ethylhexyl_phthalete), all correlated with *Ruminococcaceae_UCG-014*.

As expected, different metabolites were significantly correlated with different genera. For example, PE_16 and PE_14 were clustered with LysoPE_15 and were positively correlated with *Prevotellaceae_UCG-001, Alloprevotella, and Erysipelatoclostridium* while negatively with *Rickenella and Helicobacter*. DG-15 was negatively correlated with *unclassified_f_Lachnospiraceae* and *Anaerotruncus*. The cluster of metabolites including DL_phenylalanine, hydroxyphenethylamine 2, L-isoleucine, and piperidine was positively correlated with norank_f_Bacteroidales_S24-7-group and negatively correlated with Alistipes, Candidatus_Saccharimonas, and norank_0_Mollicutes_RF9 (Fig. 5A). Some of the 31 X-axis variables in Fig. 5A were highly correlated among each other. To reduce the impact of collinearity, we used a variable reduction approach, namely the Variance Inflation Factor (VIF) analysis. TNF-α, Bis-2-ethylhexyl_phthalat, and Triphenyl-phosphate had VIF values>10 and thereby were excluded from the subsequent Permutational Multivariate Analysis Of Variance Analysis (PERMANOVA). We identified 10 variables significantly correlated with bacterial genera (*P*<0.05) (Fig. 5B). However, only *Ruminoscoccaceae-UCG-014* was significantly associated with inflammation (IL-6), neurotransmission (5-HT), and microbial metabolism (PS).

## Discussion

Previous studies have shown that chronic stress promoted tumor invasion and metastasis by activating the hypothalamus-pituitary-adrenal axis and sympathetic nervous system 20. We found that CRS exposure increased serum levels of IL-6 and TNF-α, indicative of a CRS-induced intestinal immune-inflammatory response. Gut inflammation is one of the causes of intestinal barrier dysfunction, which can lead to an increased risk of intestinal flora ectopic 21. We also observed a neuro-inflammatory response to CRS exposure, reflected in decreased levels of circulating serotonin (5-HT) and increased levels of circulating neuroendocrine hormones (ADR and NADR). Both 5-HT and NADR are crucial neurotransmitters of mood and cognition regulation. Our finding is consistent with a previous report that the balances of both hormones (5-HT and NADR) can be disrupted by chronic stress 22. Studies have shown that depressed patients displayed a disturbance of the gut microflora, including changes in abundance of microbial metabolites 23, 24. In the CRS mouse model, we found that *Ruminococcaceae_UCG-014* was significantly more abundant in CRS-exposed mice. Hence, we speculate that *Ruminococcaceae_UCG-014* may play an important role in the process of CRS-mediated growth and metastasis of colorectal cancer.

Studies have documented a contributive role of stress-responsive neurotransmitters, such as catecholamines and 5-HT, in mediating the tumor pathogenesis. For example, Sustained activation of adrenergic signaling by catecholamines promotes tumor growth and metastasis through enhancing the production of brain-derived neurotrophic factor (BDNF) by tumor cells and BDNF-mediated tumoral innervation 25. A study by Zhu, *et al.* reported that 5-HT produced from enteric serotonergic neurons has function to initiate Wnt/β-catenin signaling in colorectal cancer stem cells (CSCs) via activating its HTR1B/1D/1F receptors, driving CSC self-renewal and colorectal tumorigenesis 26.

The positive rate of 5-HT has been shown to be increased sequentially in patients with non-atrophic gastritis, intestinal metaplasia and gastric cancer 27, 5-HT can enhance the cell viability of gastric adenocarcinoma, reduced cellular and lipid reactive oxygen species, and suppressed ferroptosis via triggering HTR2B-mediated PI3K/Akt/mTOR signaling pathway 28. In non-small-cell lung cancer, the 5-HT7 receptor, which expression is highly associated with lymph node metastasis and advanced TNM stage, stimulate the migration and invasion of lung carcinoma cells 29. In contrast to observations mentioned above that 5-HT and its receptors positively regulate tumor progression, one study revealed that the 5-HT/HTR1E signaling attenuated by chronic stress resulted in higher levels of cell proliferation and epithelial mesenchymal transition of ovarian cancer cells, thereby promoting tumor growth and peritoneal dissemination 30. 5-HT is also a regulator of angiogenesis by reducing the expression of matrix metalloproteinase 12 in tumor-infiltrating macrophages, entailing lower levels of angiostatin 31. 5-HT has additional functions to promote the maturation and migratory property of bone marrow-derived dendritic cells (DCs) via 5-HTR7 32 and the development of DCs, which shuttle 5-HT to naive T cells and thus modulate T cell proliferation and differentiation 33. Current evidence indicates that stress-responsive neurotransmitters and receptors, such as 5-HT and 5-HTRs, indeed affects tumor pathogenesis by regulating tumor cell malignancy, or interacting with immune cells within tumor microenvironment, although the action of neurotransmitters on tumor progression remains controversial.

We found that CRS exposure altered metabolite profiles of the gut microbiota. Among the 24 metabolites that showed a significant difference in abundance between CRS-exposed and control mice, carpaine and DG (15:0/20:4(5Z,8Z,11Z,14Z)/0:0) were more abundant in CRS-exposed mice. DG is catalyzed into phosphatidic acid by diacylglycerol kinases (DGK); and DGK isoforms (DGKα and DGKζ) are expressed in tumor-infiltrating T lymphocytes. In T lymphocytes, DGKα and DGKζ limit the activation of the PLCγ/Ras/ERK axis, providing a critical checkpoint to inhibit T cell responses 34. DGKα contributed to Src, p53 and Ras oncogenic functions 35, 36. Carpaine can affect the myocardium directly due to its macrocyclic dilactone structure (a possible cation chelating structure) 37. Studies have shown that carpaine has anti-thrombocytopenic and antiplasmodial biological activities 38, 39; but no research has directly linked carpaine with colorectal cancer. However, a recent study provided relevant insights by showing that expression of genes involved in amino acid-dependent acid resistance mechanisms of meta-gut Escherichia coli responded differently to acid, salt, and oxidative pressures under aerobic conditions 9.

Among the 22 metabolites showing a deceased abundance in our CRS-exposed mice, phosphatidylethanolamine (PE) and hemolytic phosphatidylethanolamine [LysoPE (15:0/0:0)] are well known for their role in cancer. Studies have shown that tumor cells can flip PE from the inner leaflet to the outer leaflet, PE deficiency induces lipid stress, increasing the risk for cancer progression 40. In tumor vascular endothelial cells, PE has been shown to form a pyrrole adduct with ophiobolin A (a sesterterpenoid), thereby killing tumor cells 41. LysoPE is formed by losing one molecule of fatty acid of PE under the decomposition of phospholipase A1 (an anticancer enzyme) 42. Hence, our finding, that CRS exposure significantly reduced the abundance of PE and LysoPE, further supports a link between CRS-altered gut microbial metabolites and CRS-enhanced tumor growth and metastasis.

The results indicated that microbial metabolites were enriched differentially in CRS-exposed mice versus control mice in the following two KEGG pathways: the 'protein digestion and absorption: OS' pathway and the 'tropane, piperidine and pyridine alkaloid biosynthesis: M' pathway. The former pathway has been associated with pulmonary metastasis of osteosarcoma 43, and vitamin digestion and absorption were confirmed to promote the progression of colon cancer 44. Moreover, this pathway was reported to be the most significantly enriched KEGG pathway in the intestine of a rat model of chronic unpredictable mild stress-induced depression 45. To the best of our knowledge, however, the latter pathway ('tropane, piperidine and pyridine alkaloid biosynthesis: M) has not been previously linked to chronic stress, cancer, or intestinal barrier. As a key player in the homeostasis of the gut microbiota, *Ruminococcaceae_UCG-014* has been connected to pathological processes for colitis, ulcerative colitis carcinogenesis and antibiotic-associated diarrhea 46-48. Changes in gut or intestinal abundance of *Ruminococcaceae_UCG-014*, by therapeutic interventions, have been associated with changes in TNF-α and IL-6 levels, gut dysbiosis, infiltration of inflammatory cells into the ileum and colon, oxidative stress levels, and tumor load 49, 50. Consistently, we found that CRS exposure altered the abundance of *Ruminococcaceae_UCG-014*, which was associated with altered microbial metabolites and increased systemic inflammation likely spilled over from intestinal tissue inflammation. Furthermore, the positive correlations we found for *Ruminococcaceae_UCG-014* with IL-6 and ADR suggest that CRS exposure may have resulted in persistent intestinal inflammation and the hypothalamus-pituitary-adrenal (HPA) axis activation via increasing the abundance of this particular bacterial genus, along with a general disruption of the gut microbiome as described earlier. Our recent study revealed that altered gut microbiome, highly enriched with *Ruminococcaceae* accompanied with a shift in tryptophan metabolism towards kynurenine production, was associated with the severity of depression in a cohort of irritable bowel syndrome (IBS) 51. Perhaps for the first time, we found a significant association between *Ruminococcaceae_UCG-014* and 5-HT in a mouse model of chronic stress. More than 90% 5-HT is synthesized in the gut, diminished production of 5-HT has been associated with depressive-like behaviors in mice 52. Studies have also shown that increased 5-HT biosynthesis in the gastrointestinal tract by certain microbes can improve gastrointestinal motility and hemostasis 53.

There remain certain study limitations that the causal relationship between chronic stress-induced gut dysbiosis and the progression of CRC is yet to be clarified, and we could not afford a study design that would allow for both between-treatment comparisons and for before-after treatment comparisons due to logistic constrains such as the difficulty in maintaining the animals for a longer duration. Future studies are recommended to adopt this double comparison design to increase the study power for causality of the findings.

## Conclusions

In the present study, we found a link between stress-induced worsening of colorectal cancer and gut microbial perturbation. A bacterial genus was specifically involved in this link. Our findings support the importance of targeting the intestinal microbiome as a novel therapeutic strategy for colorectal cancer. Our findings suggest that mitigating stress-induced disruption of *Ruminococcaceae_UCG-014* may help alleviate the progression of this common cancer.
